# The cardiovascular toxicity of polystyrene microplastics in rats: based on untargeted metabolomics analysis

**DOI:** 10.3389/fphar.2024.1336369

**Published:** 2024-05-10

**Authors:** Zikai Song, Haidi Wu, Xiaoqi Fang, Xuemin Feng, Liting Zhou

**Affiliations:** ^1^ Department of Cardiology, The First Hospital of Jilin University, Changchun, China; ^2^ Department of Occupational and Environmental Health, School of Public Health, Jilin University, Changchun, China; ^3^ Department of Neurology, The First Hospital of Jilin University, Changchun, China

**Keywords:** polystyrene microplastics, cardiovascular toxicity, non-targeted metabolomics, inflammatory response, oxidative stress

## Abstract

**Background:**

Polystyrene microplastics (PS-MPs) exhibit multi-target, multi-dimensional, chronic, and low toxicity to the cardiovascular system. They enter the bloodstream through the gastrointestinal tract and respiratory system, altering blood parameters and conditions, inducing thrombotic diseases, and damaging myocardial tissue through the promotion of oxidative stress and inflammatory responses in myocardial cells. However, many of the links and mechanisms remain unclear.

**Methods:**

In this study, 48 wistar rats were randomly divided into four groups and exposed to different concentrations of PS-MPs: control group (0 mg/kg/d), low dose group (0.5 mg/kg/d), middle dose group (5 mg/kg/d) and high dose group (50 mg/kg/d), with 12 rats in each group. After 90 consecutive days of intragastric administration of PS-MPs, biochemical markers in myocardium, aorta and blood were detected, and HE staining was performed to observe the toxic effects of PS-mps on cardiovascular system. Furthermore, non-targeted metabolomics methods were used to analyze the effect of PS-MPs exposure on the metabolism of cardiovascular system in rats, and to explore its potential molecular mechanism.

**Results:**

The results revealed no pathological changes in the heart and aorta following PS-MPs exposure. However, the myocardial enzyme levels in the high dose PS-MPs group of rats showed a significant increase. Moreover, exposure to polystyrene microplastics caused a disorder in lipid metabolism in rats, and led to an increase in indicators of inflammation and oxidative stress in myocardial and aortic tissues, but resulted in a decrease in the level of IL-6. Untargeted metabolomics results showed that metabolites with antioxidant and anti-inflammatory effects, including equol and 4-hydroxybenzoic acid, were significantly upregulated.

**Conclusion:**

These results suggest that long-term exposure to high concentrations of PS-MPs may lead to abnormal lipid metabolism and cardiovascular system damage. The mechanism may be related to oxidative stress and inflammatory response. Exogenous antioxidants and changes in own metabolites may have a protective effect on the injury. Therefore, understanding the toxicological mechanism of PS-MPs not only helps to elucidate its pathogenesis, but also provides new ideas for the treatment of chronic diseases.

## 1 Introduction

Plastics are widely used because of their simple, durable and plastic characteristics. However, with the increase of the consumption, non-degradation, waste management and other problems make it become one of the major environmental threats. Plastic pollution is not only a prominent threat to the environment, but studies have found that smaller pieces of plastic broken down by ultraviolet light can enter organisms and threaten human health (Conti et al., 2021). Among them, plastic fragments, fibers or particles with a diameter less than 5 mm are called microplastics, which widely exist in the ocean, soil and other environmental media, and can also release certain harmful gases through their own oxidative decomposition, causing air pollution (Klein and Fischer, 2019; Herrera et al., 2022). According to the results of a survey conducted in 2019 in the United States of America (Cox et al., 2019), the average annual intake of microplastics per person through diet is estimated to be 39,000 to 52,000 particles, which increases to an estimated 74,000 and 121,000 particles per year when inhalation is also taken into account. Different polymer types of microplastics includes polyethylene, polypropylene, polystyrene, polyamide, nylon, and rayon, etc (Shruti et al., 2021).

Polystyrene microplastics (PS-MPs) are widely used in construction products, plastic packaging, personal care products and food containers (such as toothpaste, cosmetics and cups) due to their high transparency, wear resistance and easy dyeing (Schwabl et al., 2019). In the environment, PS-MPs is widely found in atmospheric, water, soil and other environmental media, and which can penetrate the food web through aquatic organisms, animals and humans (Nanda et al., 2022).

Medical studies have found that PS-MPs can be absorbed by organisms and accumulate in the body, and affect human health through the food chain. Microplastics, especially nanoplastics with smaller diameters, can enter the lungs through the respiratory tract. Gastrointestinal absorption is another way that microplastics affect human health. Through gastrointestinal uptake, microplastics can enter blood, and distribute to liver, muscle, brain and heart (Yong et al., 2020). *In vitro* and *in vivo* studies in animals have shown that potential inhalation or ingestion of microplastics can cause a variety of biological effects, including physical (particle) toxicity, triggering oxidative stress, cytokine secretion, cell damage, inflammatory and immune responses, as well as DNA damage, neurotoxicity and lipid metabolic effects (Deng et al., 2017; Schirinzi et al., 2017; Deng et al., 2018; Lim et al., 2019; Vethaak and Legler, 2021).

The cardiovascular system, respiratory system, and digestive system share similar attributes and serve as both carriers for the migration of microplastics within the body and targets of microplastic damage. Therefore, the impact of microplastics on the cardiovascular system has gained extensive attention. Most of the previous studies focused on Marine organisms and mammals. Starting from the detection of microplastic fragments in fish gills (Koongolla et al., 2020), it has been confirmed that microplastics not only enter the bloodstream and alter blood parameters and conditions but also induce thrombotic diseases. They can also act on myocardial tissue, leading to myocardial damage through the promotion of oxidative stress and inflammatory reactions in myocardial cells. Yue et al. applied 5 μm PS-MPs of different concentrations to chicken hearts and primary cardiomyocytes and found severe pathological damage and ultrastructural changes in the heart, as well as myocardial hypothermia, inflammatory cell infiltrations, and mitochondrial damage (Zhang et al., 2022). PS-MPs caused abnormal levels of antioxidant enzymes and excessive production of reactive oxygen species, resulting in changes in the NF-κB-NLRP3-GSDMD and AMPK-PGC-1α pathways, leading to oxidative stress, myocardial hypothermia, inflammation, mitochondrial dysfunction, and energy metabolism disorders (Zhang et al., 2022). Additionally, Li et al. confirmed in a rat model that exposure to PS-MPs leads to structural damage and apoptosis of myocardial cells, as well as collagen proliferation in the heart. The mechanisms may involve PS-MPs activating the Wnt/β-catenin pathway and triggering oxidative stress-induced myocardial cell apoptosis, ultimately inducing myocardial fibrosis and causing cardiovascular toxicity (Li et al., 2020). A recently published clinical observational study found that microplastics and nano-microplastics are present in human carotid plaques, and patients with microplastics and polystyrene microplastics detected within atherosclerotic plaques had a significantly higher risk of primary endpoint events such as myocardial infarction and stroke than patients without these substances detected (Marfella et al., 2024). Based on the current literature, PS-MPs has low toxic damage to the cardiovascular system, and it is a multi-target, multi-dimensional and chronic effect. Oxidative stress and inflammation may be the main toxicological mechanism. However, many links and mechanisms need to be further explored and clarified.

It has been discovered that there is a close correlation between the metabolites produced during the process of metabolism and the occurrence of diseases. Metabolites, as direct indicators of biochemical activity, are more easily related to phenotypes. They serve as substrates and products for driving basic cellular functions such as energy production and storage, signal transduction, and cell apoptosis. In addition to being directly produced by the body, metabolites can also be obtained from microorganisms, diet, and other external sources (Johnson et al., 2012). Metabolomics, as a powerful method, has been widely used in scientific research and clinical diagnosis, helping us explore and understand diseases. Non-targeted metabolomics detection based on LC-MS can comprehensively detect metabolites and is widely used in evaluating the components of environmental pollution and the mechanisms of bodily injury. In the case of microplastic-induced bodily injury, it can not only evaluate its direct damage targets, but also comprehensively evaluate the molecular mechanisms of microplastic-induced bodily injury. Microplastic-induced bodily injury has the characteristics of chronicity and accumulation. Long-term accumulation can cause changes in bodily metabolites, driving cellular energy production and storage, signal transduction, and cell apoptosis. Therefore, this study utilized a non-targeted metabolomics approach to investigate the effects and molecular mechanisms of PS-MPs exposure on the cardiovascular system of rats.

## 2 Methods

### 2.1 Animal subjects and grouping

Forty-eight 6-week-old SPF Wistar male rats, weighing (180 ± 20) g, were selected. All rats were provided by Beijing Huafukang Biotechnology Co., LTD., and raised in the Medical Animal Experimental Center, School of Public Health, Jilin University. After 1 week of adaptive feeding, the rats were randomly divided into four groups according to the concentration of PS-MPs: the control (0 mg/kg/d) group, low dose (0.5 mg/kg/d) group, middle dose (5 mg/kg/d) group and high dose (50 mg/kg/d) group, with 12 rats in each group. The rats were exposed by gavage once a day for 90 days. The low, middle and high dose groups were given the corresponding concentration of 0.5 μm PS-MPs, which was thoroughly mixed by ultrasound 30min before exposure, and the control group was given the corresponding volume of distilled water. The status of rats was observed every day, and the changes in body weight, diet and water intake of rats were recorded. The exposed doses of PS-MPs were selected based on the cardiotoxicity studies of PS-MPs in rats (Li et al., 2020; Wei et al., 2021).

All rats were fed in the Medical Animal Experiment Center of the School of Public Health, Jilin University. This study was approved by the Ethics Committee of School of Public Health, Jilin University (No.: 2022-07-08).

### 2.2 Tissue sample collection

Twenty-four hours after the last exposure, the rats were fasted for 12 h. After anesthesia, blood samples were collected from the heart, and then the heart and thoracoabdominal aortic tissue were carefully dissected out. The blood samples were left at 4 °C for 1 h and then centrifuged at 3500rpm for 15 min to obtain the upper layer for the detection of blood parameters and non-targeted metabolomics tests. After the organ tissues were thoroughly cleaned with normal saline, the surface moisture was blotted with clean filter paper and weighed. A portion of the tissue was fixed in 4% paraformaldehyde for HE staining of pathological sections. The remaining tissue was immediately placed into cryopreserved tubes and placed in liquid nitrogen, and 24 h later transferred to −80 °C refrigerator for storage.

### 2.3 Detection of myocardial tissue and serum parameters

Cardiac troponin T (cTNT), creatine phosphokinase isoenzyme (CK-MB), lactate dehydrogenase (LDH), interleukin-1 (IL-1), interleukin-1β (IL-1β), intercellular adhesion molecule-1 (ICAM-1), superoxide dismutase (SOD), glutathione peroxidase (GSH), tumor necrosis factor-α (TNF-α), interleukin-18 (IL-18) and interleukin-6 (IL-6) in myocardial tissue were detected by ELISA. Serum triglyceride (TG), total cholesterol (TC), low density lipoprotein (LDL), high density lipoprotein (HDL) and glucose (GLU) levels were detected with commercial kits (Nanjing Jiancheng Bioengineering Institute, China). Serum malondialdehyde (MDA), GSH, IL-1, TNF-α, SOD and acid phosphatase (ACP) activity were detected by ELISA. These experimental index kits were purchased from the Nanjing Jiancheng Institute of Biotechnology (Nanjing, China). All experiments were performed strictly according to the manufacturer’s instructions.

### 2.4 Histopathological analysis

The tissues of all groups were also fixed in 4% paraformaldehyde, embedded in paraffin wax, sectioned at 4 μm thickness, and stained with hematoxylin and eosin (H&E) for microscopic observation.

### 2.5 Non-targeted metabolomics

#### 2.5.1 Sample preparation/metabolite extraction

Melt all samples at 4 °C. Take 100 μL from each sample in a 2 mL microcentrifuge tube (for samples ≤50μL, the experimental system is halved for extraction, while the residual system remains unchanged). Add 400 μL of pre-cooled methanol (−20 °C) to each microcentrifuge tube, shake for 60 s. Centrifuge at 12,000 rpm at 4 °C for 10 min. Take the total supernatant and transfer it to a new 2 mL centrifuge tube. Concentrate and dry it *in vacuo*. Dissolve 150 μL of 2-chlorophenylalanine (4 ppm) in 80% methanol solution and filter the supernatant using a 0.22 μm membrane to obtain the sample to be measured. Take 20 μL from each sample to be measured and mix it into a QC sample (QC: quality control, used to correct the deviation of the analysis results of mixed samples and errors caused by the analytical instrument itself). Perform LC-MS testing with the remaining sample to be measured. The datasets presented in the study can be found in Metabolights database (accession number: MTBLS9366, URL: www.ebi.ac.uk/metabolights/MTBLS9366).

#### 2.5.2 LC-MS detection

Chromatographic conditions: ① Instrument: U3000, Thermo. ② Column: ACQUITY UPLC^®^ HSS T3 1.8 µm (2.1 × 150 mm) chromatographic column. ③ Chromatographic separation conditions: column temperature 40 °C; flow rate 0.25 mL/min; mobile phase as follows: positive ion 0.1% formic acid water (C)-0.1% formic acid acetonitrile (D); negative ion 5 mM ammonium formate water (A)-acetonitrile (B); injection volume 2 μL; auto sampler temperature 8 °C. ④The gradient elution program of the mobile phase is as follows: 0-1 min, 2% B/D; 1-9 min, 2%–50% B/D; 9-12 min, 50%–98% B/D; 12-13.5 min, 98% B/D; 13.5-14 min, 98%–2% B/D; 14-20 min, 2% D in positive mode (14-17 min, 2% B in negative mode).

Mass Spectrometry Conditions: ①Instrument: Q Exactive HF-X, Thermo; ②Mass Spectrometry Conditions: Electrospray ionization source (ESI), positive and negative ionization modes, positive ion spray voltage of 3.50 kV, negative ion spray voltage of 2.50 kV, sheath gas 30 arb, auxiliary gas 10 arb. Capillary temperature 325°C, with a resolution of 60,000 for full scan, scanning range 81–1,000, and using HCD for second-stage fragmentation, collision voltage of 30 eV, while employing dynamic exclusion to remove unnecessary MS/MS information.

### 2.6 Data processing

The original data were converted into mzXML format (XCMS input file format) by Proteowizard software (V3.0.8789). Peaks identification, peak filtration and peak alignment were performed using the XCMS package of R (V3.3.2). The main parameters were BW = 2, PPM = 15, Peakwidth = C (5, 30), mZWId = 0.015, MZDIff = 0.01, Method = centWave. The data matrix including mass to charge ratio (m/z), retention time (rt) and intensity was obtained. In positive ion mode, 13,628 precursor molecules were obtained. A total of 14,889 precursor molecules were obtained in negative ion mode, and the data were exported to Excel for subsequent analysis. Batch normalization of peak areas was used to allow comparison of data of different magnitudes.

First, the Base peak chromatogram (BPC) was obtained. QC samples were inserted into the test samples before, during and after injection to obtain the data information of QC samples. The stability of the system during the whole experiment was controlled and the correlation analysis of samples was performed to obtain the PCA analysis chart. On the basis of quality control (QC), quality assurance (QA) was further performed to delete the features with poor repeatability in QC samples and obtain a higher quality dataset. In QC samples, RSD (relative standard deviation) and LT; The proportion of characteristic peaks of 30% can reach about 70%, indicating that the data are good. The metabolites were further analyzed by principal component analysis (PCA), partial least squares discriminant analysis (PLS-DA), and orthogonal partial least multiplication-discriminant analysis (OPLS-DA).

By screening the metabolites, differential metabolites (biomarkers) were found. The screening criteria for relevant differential metabolites were: 1) *p*-value ≤0.05 and VIP ≥1; 2) *p*-value ≤0.05 and fold change ≥1.5 or ≤0.667; 3) one-way ANOVA *p*-value ≤0.05; 4) two-way ANOVA *p*-value ≤0.05. The identification of metabolites begins with the confirmation of the exact molecular weight of the metabolite (molecular weight error <30 ppm), according to MS/MS pattern after the pieces of information in HumanMetabolome Database (HMDB) (http://www.hmdb.ca), Metlin (http://metlin.scripps.edu), Massbank (http://www.massbank.Jp/), LipidMaps (http://www.lipidmaps.org), mZclound (https://www.mzcloud.org), And further matching annotations in the self-established standard database to obtain accurate metabolite information. The results of screening differential metabolites were further visualized in the form of volcano plot, and differential metabolite z-score analysis, differential metabolite hierarchical clustering analysis (HeatMAP), and differential metabolite association analysis were performed. KEGG (Kyoto Encyclopedia of Genes and Genomes) pathway analysis was used to analyze the pathways of differential metabolites.

### 2.7 Statistical analysis

All the continuous variables in myocardial tissue and blood indicators were expressed as mean ± standard deviation ( $\bar{x}$ ± *SD*) and compared with the use of analysis of variance, with the use of the LSD test for further pairwise comparisons. Multiple testing was performed. Non-normally distributed variables in myocardial tissue and blood indicators were expressed as *M* (*Q*_*25*_, *Q*_*75*_), and the rank sum test was used between groups. *p* values <0.05 in double-tailed were considered to be statistically significant between the two groups. The error diagram of biomarker intensity and the above statistical analysis were implemented by IBM SPSS 24.0.

## 3 Results

### 3.1 Effects of PS-MPs exposure on myocardial tissue in rats

#### 3.1.1 Myocardial HE staining

**FIGURE 1:**
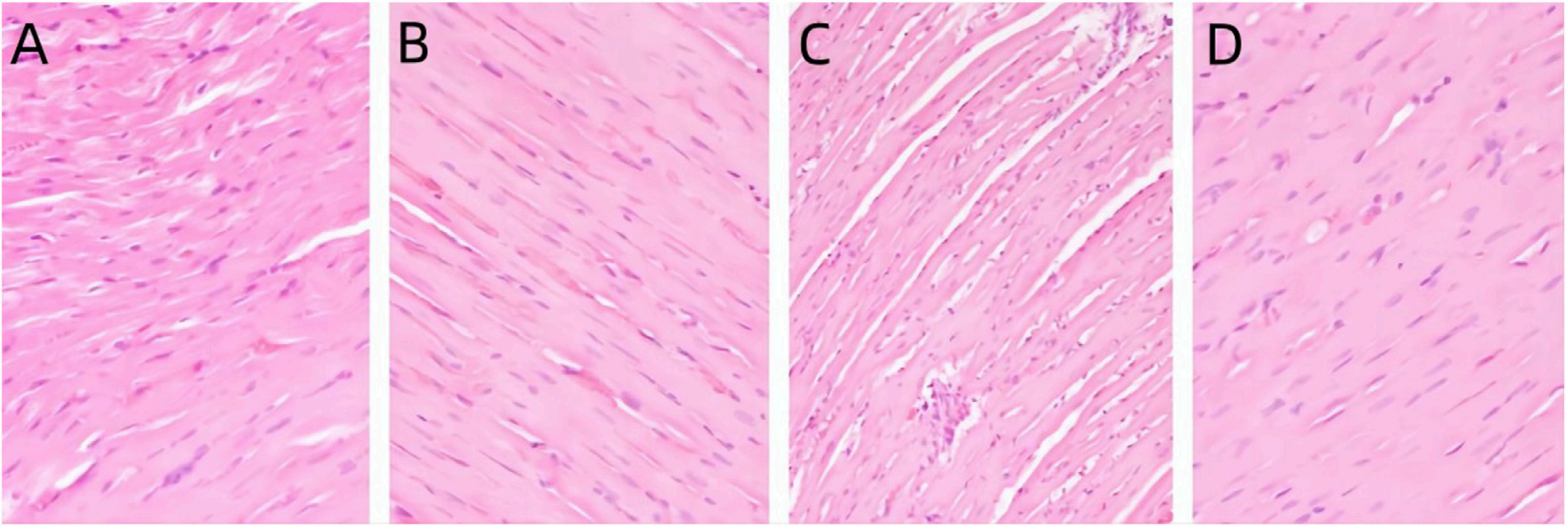
Pathological changes of heart in rats exposed to PS-MPs. **(A)** 0 mg/kg/d group, **(B)** 0.5 mg/kg/d group, **(C)** 5 mg/kg/d group, **(D)** 50 mg/kg/d group.

In the control group, the cytoplasm of cardiomyocytes was red stained, the intercellular connection was tight, and there was no myocardial interstitial edema or abnormal inflammatory reaction in the myocardial interstitium. Compared with the control group, there was no significant difference in the morphology of cardiomyocytes in each treatment group, as shown in Figure 1.

#### 3.1.2 Changes of myocardial tissue damage and inflammatory markers in rats after PS-MPs exposure

**FIGURE 2:**
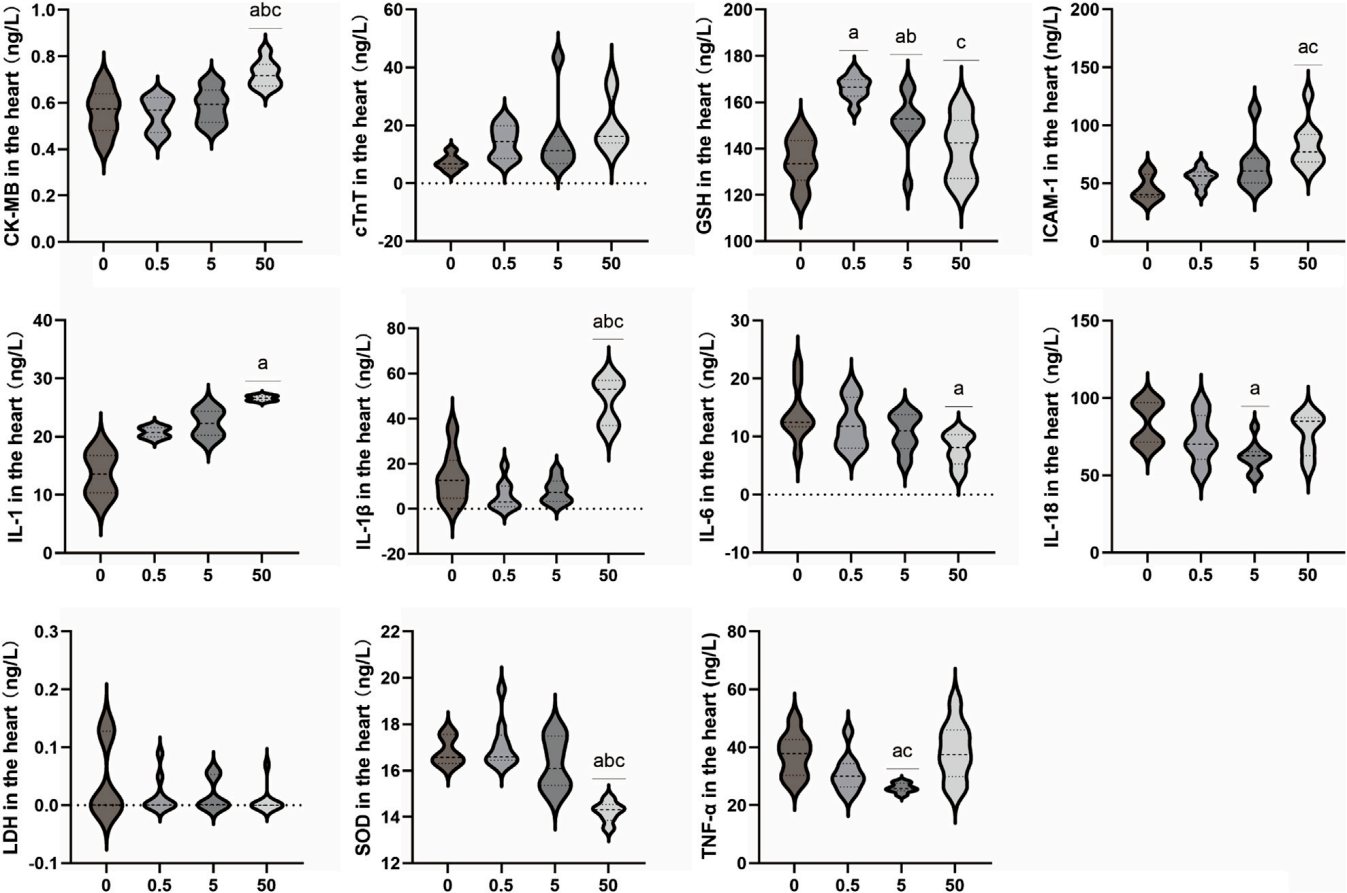
Effects of PS-MPs exposure on inflammatory markers in rat myocardial tissue. ^a^ *p* < 0.05, Comparison with 0 mg/kg/d; ^b^ *p* < 0.05, Comparison with 0.5 mg/kg/d; ^c^ *p* < 0.05, Comparison with 5 mg/kg/d.

As shown in Figure 2, the levels of cTnT in myocardial tissue homogenates of rats in each exposure group demonstrated an increasing trend, but no significant difference was observed between the exposure groups and the control group (*p* > 0.05). The level of CK-MB was significantly higher in the 50 mg/kg/d group compared to the other groups (*p* < 0.05). There were no significant differences in LDH levels in myocardial tissue homogenate between the exposed groups and the control group (*p* > 0.05). The levels of IL-1, IL-1β, and ICAM-1 in myocardial tissue increased with increasing doses of PS-MPs. The levels of IL-1, IL-1β, and ICAM-1 in the 50 mg/kg/d PS-MPs group were significantly higher than those in the control group (*p* < 0.05). In contrast, the level of IL-6 in the hearts of rats showed a downward trend. IL-6 in the 50 mg/kg/d group was significantly lower than that of the control group (*p* < 0.05). With increasing doses of PS-MPs, the levels of myocardial IL-18 and TNF-α initially decreased and then increased. Notably, the levels of myocardial IL-18 and TNF-α in the 5 mg/kg/d PS-MPs group were significantly lower than those in the control group (*p* < 0.05). Additionally, the level of TNF-α in the 50 mg/kg/d group was significantly higher than that in the 5 mg/kg/d group (*p* < 0.05). In addition, the levels of SOD in the exposure group showed a downward trend. The level of SOD in the 50 mg/kg/d group was significantly lower than that in the other groups (*p* < 0.05). The level of GSH in the group receiving 0.5 mg/kg/d was significantly higher than in the control group (*p* < 0.05). The GSH level in the group receiving 5 mg/kg/d was significantly higher than that in the control group but significantly lower than in the 0.5 mg/kg/d group (*p* < 0.05). Furthermore, the GSH level in the group receiving 50 mg/kg/d was significantly lower than in the 0.5 mg/kg/d group (*p* < 0.05).

### 3.2 Effects of PS-MPs exposure on aorta and serum parameters in rats

#### 3.2.1 HE staining of aorta

**FIGURE 3:**
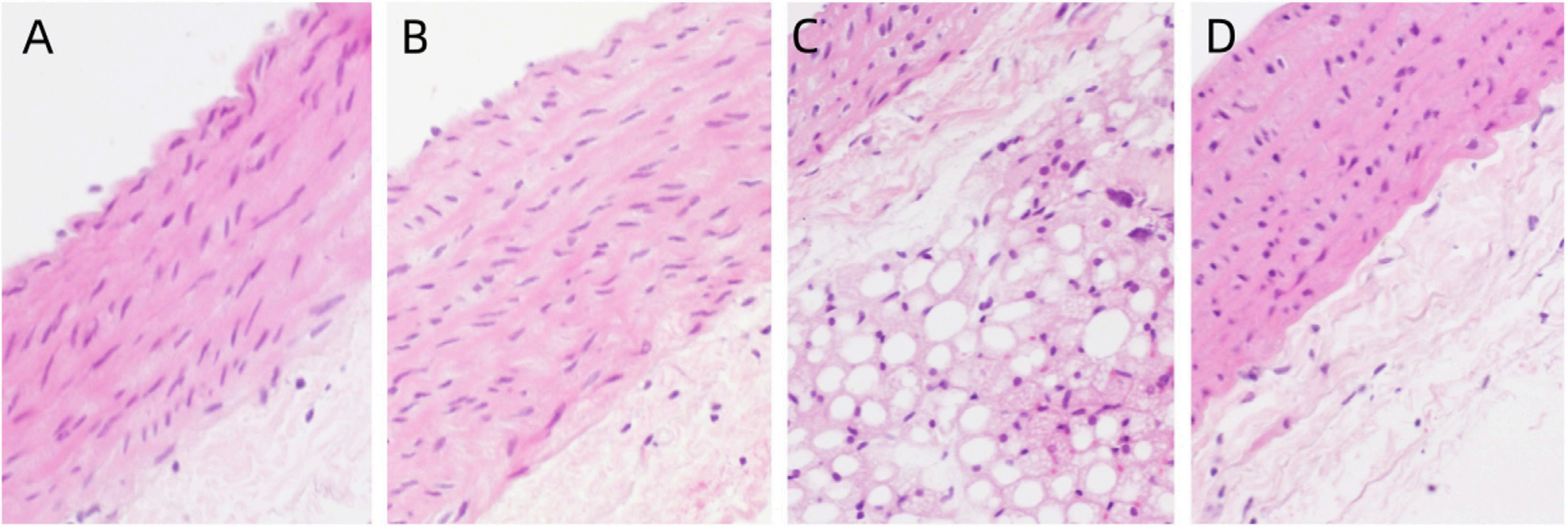
Pathological changes of aorta in rats exposed to PS-MPs. **(A)** 0 mg/kg/d group, **(B)** 0.5 mg/kg/d group, **(C)** 5 mg/kg/d group, **(D)** 50 mg/kg/d group.

The distribution of aortic endothelial cells in the control group is continuous, without any abnormal swelling or loss of endothelial cells. The aortic media is composed of multiple layers of elastic fibers, without any edema or degeneration of the elastic fibers in the aortic media. No abnormal inflammatory response is observed in the aortic adventitia, indicating a normal aortic morphology. Compared to the control group, there were no morphological differences observed in the exposed groups with different concentrations of PS-MPs. (Figure 3).

#### 3.2.2 Effects of PS-MPs on lipid and blood glucose level in rats

**FIGURE 4:**
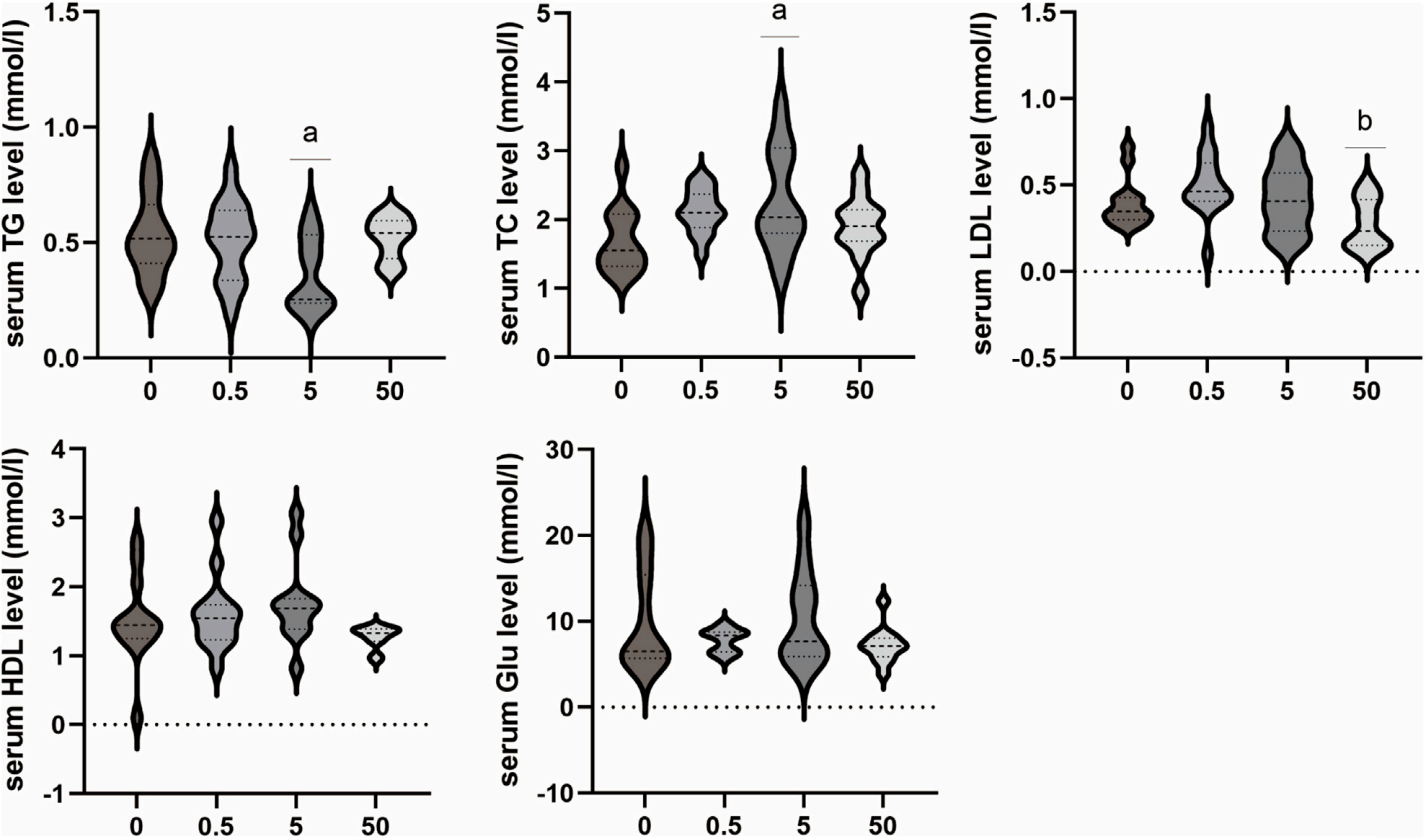
Effects of PS-MPs on lipid and blood glucose level in rats. ^*^ *p* < 0.05, Comparison with 0 mg/kg/d; ^**^ *p* < 0.05, Comparison with 0.5 mg/kg/d.

The results demonstrated that exposure to PS-MPs had no impact on blood glucose. However, the concentration of exposure affected the levels of LDL, TC, and TG in blood lipids. As the PS-MPs exposure dose increased, LDL levels exhibited an initial increase followed by a decrease. The serum LDL level in the 50 mg/kg/d group was significantly lower than that in the 0.5 mg/kg/d group (*p* < 0.05). The TC level demonstrated a rising trend, and the 5 mg/kg/d group exhibited a significantly higher TC level compared to the control group (*p* < 0.05). In contrast, the TG level in the 5 mg/kg/d group was lower than that in the control group (*p* < 0.05). No significant differences were observed in the other lipid levels and Glu level among all groups (Figure 4).

#### 3.2.3 Effect of PS-MPs exposure on serum marker levels in rats

**FIGURE 5:**
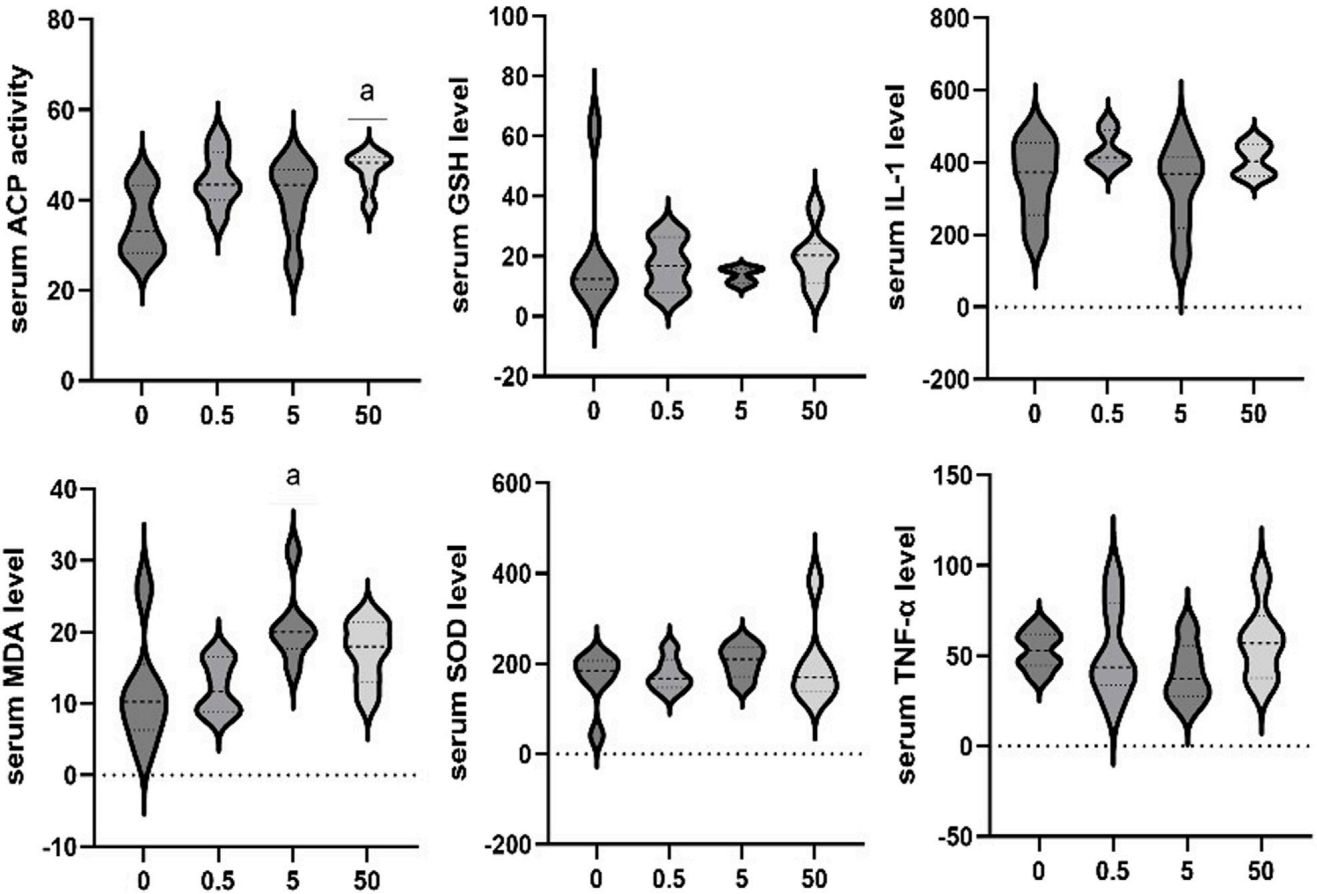
Effect of PS-MPs exposure on serum marker levels in rats. ^*^ *p* < 0.05.

The effects of exposure to different concentrations of PS-MPs on serum markers in rats were additionally assessed. The serum ACP activity of the 50 mg/kg/d group was higher than that of the control group (*p* < 0.05). Serum MDA levels of the 5 mg/kg/d group were significantly higher than that in the control group (*p* < 0.05). No significant differences were observed in other biomarkers among all groups (Figure 5).

### 3.3 Metabolomics changes in rats exposed to PS-MPs

**FIGURE 6:**
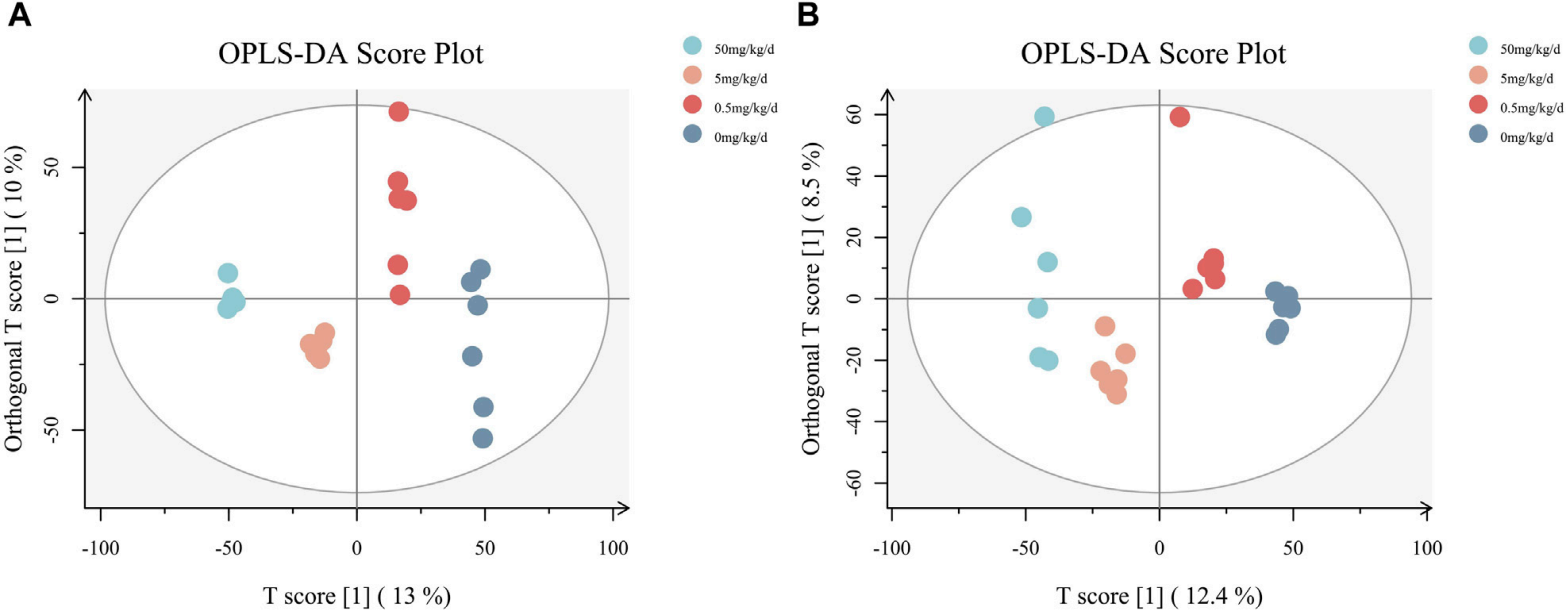
OPLS-DA diagrams for all groups. **(A)** Results of the analysis in negative ion mode, **(B)** results of the analysis in positive ion mode.

Comparisons by OPLS-DA of the metabolomic changes after exposure to different doses of PS-MPs are shown in Figure 6. The 0.5 mg/kg/d group, 5 mg/kg/d group and 50 mg/kg/d group can all be distinctly distinguished from the 0 mg/kg/d group. In general, the OPLS-DA model indicated that the metabolic profiles of rats changed after being exposed to different doses of PS-MPs.

**FIGURE 7:**
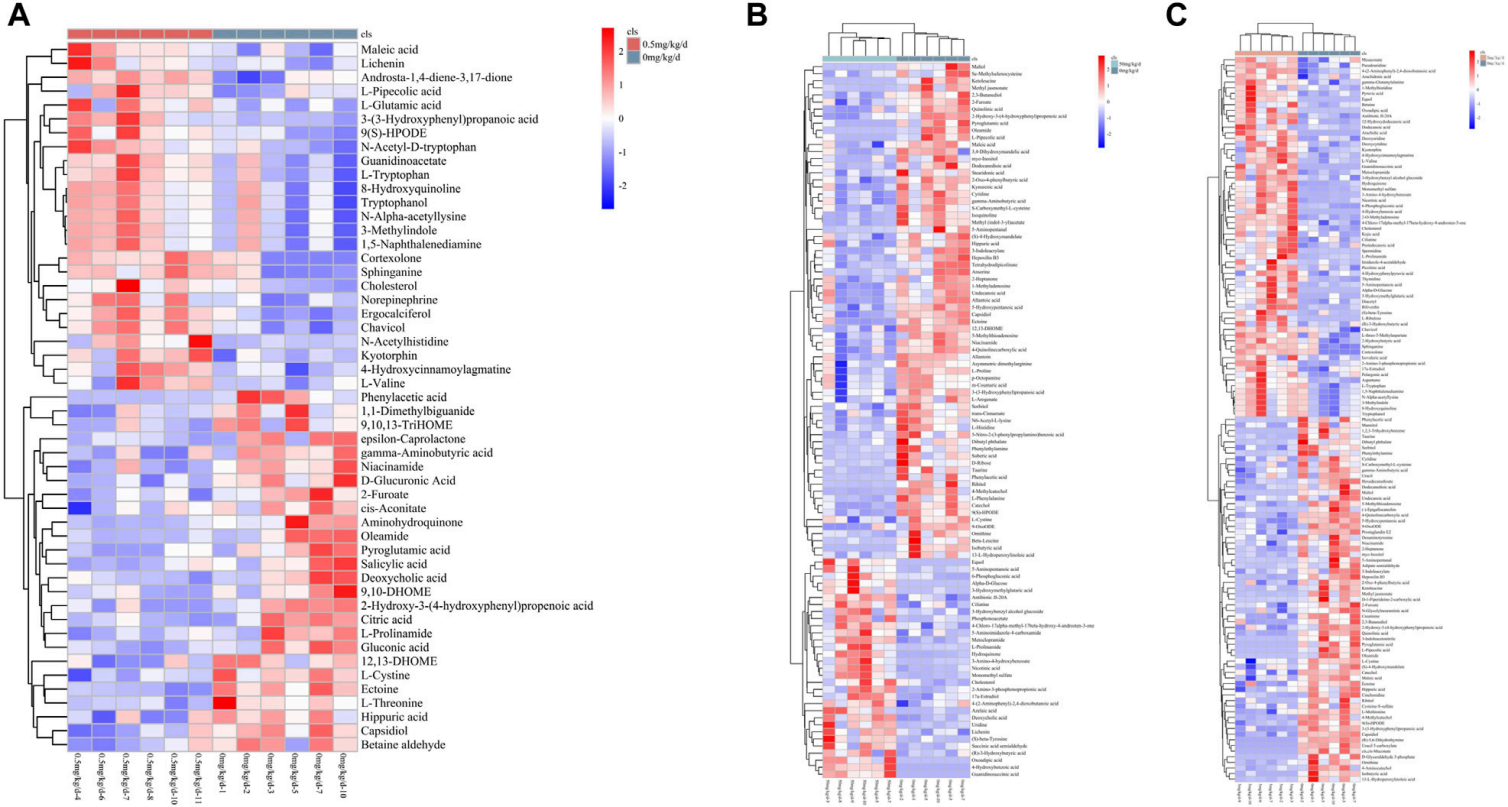
Heat map of differential metabolites between rats in different PS-MPs exposure concentration groups and the control group. **(A)** 0.5 mg/kg/d group VS. 0 mg/kg/d group, **(B)** 5 mg/kg/d group VS. 0 mg/kg/d group and **(C)** 50 mg/kg/d group VS. 0 mg/kg/d group.

As shown in Figure 7, a total of 231 metabolites with significant differential expression were identified by metabolite screening (*VIP* > 1 and *p* < 0.05). 51 significantly different metabolites were found in the 0.5 mg/kg/d group compared with the 0 mg/kg/d group, 129 significantly different metabolites were found in the 5 mg/kg/d group compared with the 0 mg/kg/d group, and 101 significantly different metabolites were found in the 50 mg/kg/d group compared with the 0 mg/kg/d group.

Figure 7 Heat map of differential metabolites between rats in different PS-MPs exposure concentration groups and the control group. (A) 0.5 mg/kg/d group VS. 0 mg/kg/d group, (B) 5 mg/kg/d group VS. 0 mg/kg/d group and (C) 50 mg/kg/d group VS. 0 mg/kg/d group.

**FIGURE 8:**
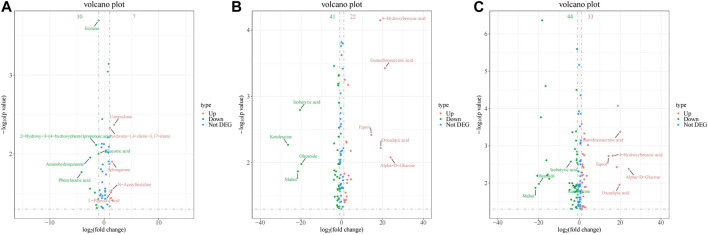
Volcano of differential metabolites between rats in different PS-MPs exposure concentration groups and the control group. **(A)** 0.5 mg/kg/d group VS. 0 mg/kg/d group, **(B)** 5 mg/kg/d group VS. 0 mg/kg/d group and **(C)** 50 mg/kg/d group VS. 0 mg/kg/d group.

The volcano diagram in Figure 8 illustrates that there were significant changes in the expression of several metabolites when the exposure concentration of PS-MPs increased. Equol, 4-hydroxybenzoic acid (4-HBA), oxoadipic acid, guanidinosuccinic acid and alpha-D-glucose were upregulated significantly. Ketoleucine, maltol, isobutyric acid and oleamide were downregulated significantly.

### 3.4 Differential metabolite pathway analysis

**FIGURE 9:**
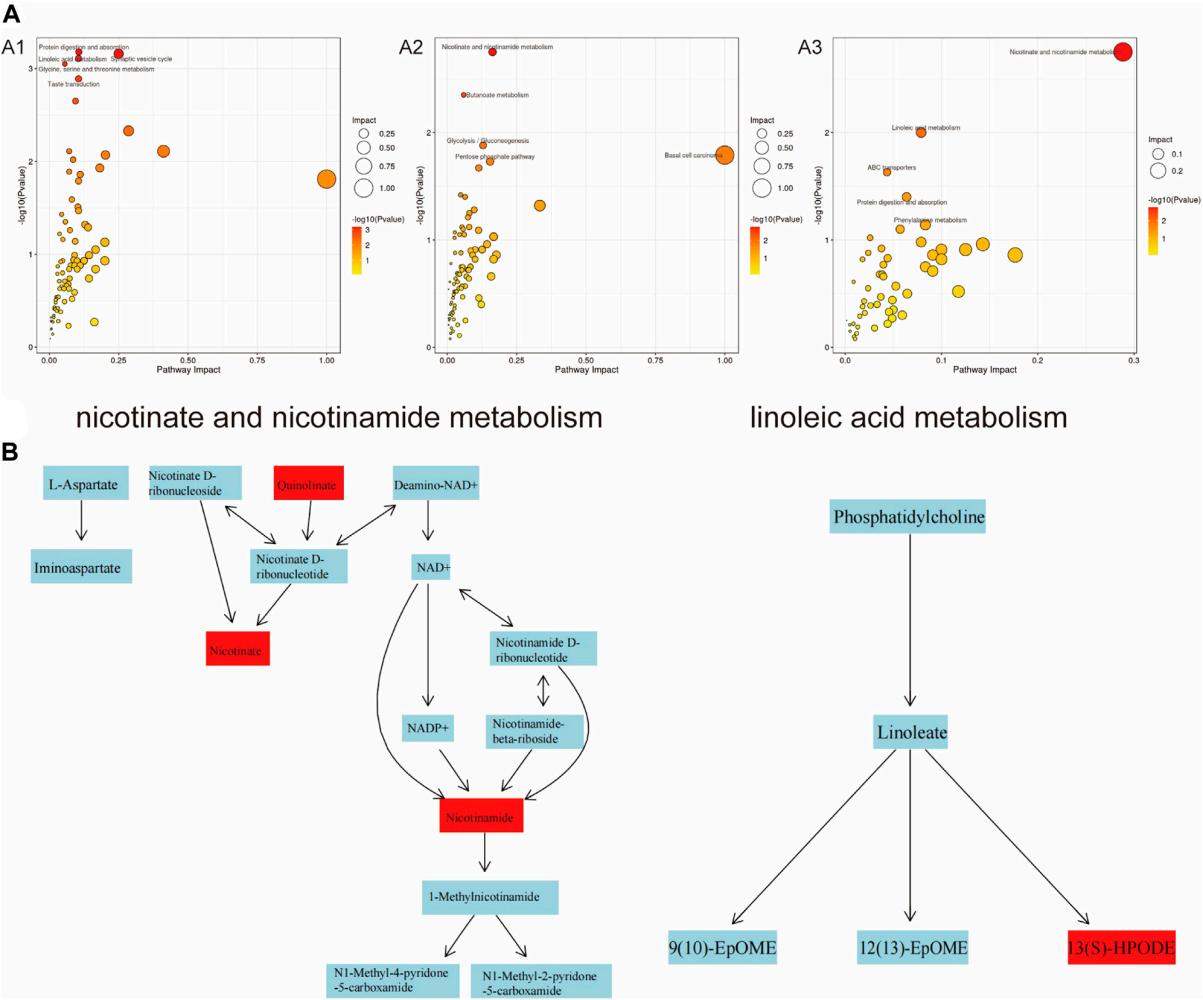
Analysis of significantly dysregulated metabolic pathways under PS-MPs exposure using MetaboAnalyst. **(A)** The color and the size of circles represent the p-value (dark colour considered significantly impacted) and the hits of metabolites in the pathway, respectively. A1: 0.5 mg/kg/d group VS. 0 mg/kg/d group, A2: 5 mg/kg/d group VS. 0 mg/kg/d group, A3: 50 mg/kg/d group VS. 0 mg/kg/d group. **(B)** Nicotinate and nicotinamide metabolic pathways and linoleic acid metabolic pathways.

Further analysis of the KEGG results revealed that “protein digestion and absorption, linoleic acid metabolism, butanoate metabolism, basal cell carcinoma” were all present in the three groups with different concentrations of PS-MPs exposure (Figure 9A). However, with the increase of PS-MPs concentration, the metabolites involved in “ABC transporter, β-alanine metabolism, phenylalanine, nicotinate and nicotinamide metabolism, lysine degradation” changed. The metabolic pathways of “nicotinate and nicotinamide” and “linoleic acid” were more active in rats exposed to high dose PS-MPs (50 mg/kg/d). Figure 9B shows that the metabolites quinolinic acid, niacin and nicotinamide in the nicotinate and nicotinamide metabolic pathways were significantly different, and the metabolite 13(S)-HPODE in the linoleic acid metabolic pathway was significantly different.

### 3.5 Correlation analysis of serum parameters and differential metabolites

**FIGURE 10:**
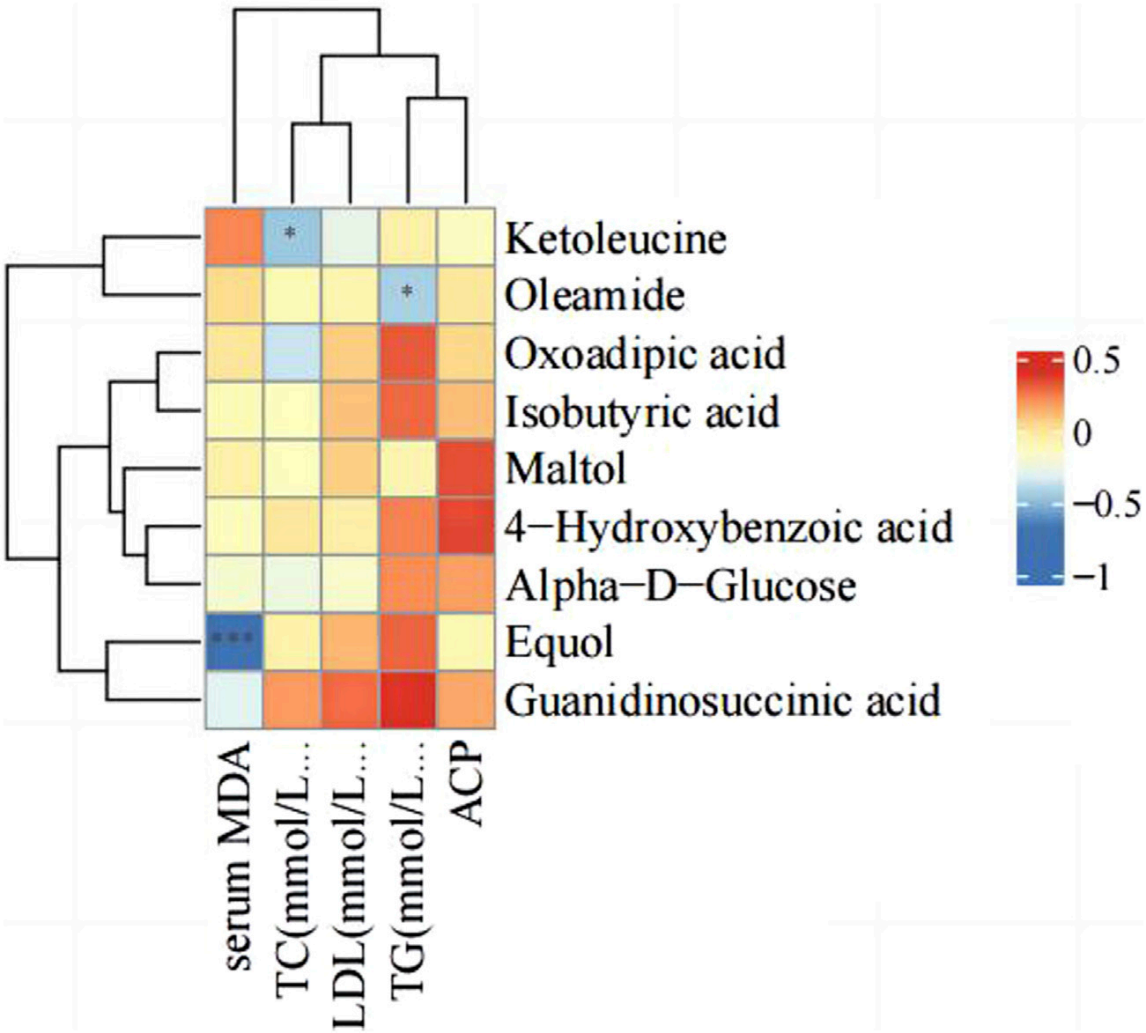
Correlation analysis of serum parameters and differential metabolites.

There were changes in LDL, TC, TG, serum ACP activity and MDA level after PS-MPs exposure. Therefore, the correlation between the above indexes and differential metabolites was analyzed in the high concentration PS-MPs exposure group (50 mg/kg/d). Figure 10 showed a negative correlation between equol and MDA levels.

## 4 Discussion

In the past 5 years, the harm of microplastic pollution to the environment and organisms has received increasing attention. According to a survey conducted in the United States in 2019, it was estimated that the average annual intake of microplastics through the diet ranges from 39,000 to 52,000 particles per person (Cox et al., 2019). If inhalation is also taken into consideration, these estimates would increase to 74,000 and 121,000 particles per year (Cox et al., 2019). However, given the escalating levels of pollution and incomplete dietary data, the actual numbers are likely to be even higher. Multiple studies have confirmed that PS-MPs can penetrate the blood barrier and reach various organs and tissues in the body, leading to chronic obstructive pulmonary disease, tumors, neurological damage, liver toxicity, kidney toxicity, reproductive toxicity, teratogenicity, ocular toxicity, and cardiac toxicity. Both PS-MPs and nanoplastics have been found to cause damage to cardiomyocytes in marine and terrestrial organisms (Lett et al., 2021; Wei et al., 2021; Wu et al., 2022; Zhou et al., 2023), and can even lead to myocardial fibrosis (Li et al., 2020; Lin et al., 2022). However, in terrestrial organisms such as chickens, rats, and mice, the main effect is localized and subtle damage to the myocardium.

Based on previous research results, this study utilized three different concentrations (0.5, 5, 50 mg/kg/d) of PS-MPs to induce toxicity in rats and extended the period of exposure to 90 days, in order to observe the impact of PS-MPs on the cardiovascular system of rats. The histopathological results of this study showed no significant pathological changes in myocardial cells and blood vessels in rats after long-term poisoning with high concentrations of PS-MPs. There was no notable increase in the levels of cardiac structural protein TNT, while the levels of CK-MB in the high-dose PS-MPs group (50 mg/kg/d) showed significant elevation. The toxic effect of PS-MPs on myocardium is characterized by chronic and low toxicity. Long-term high-dose accumulation may further lead to myocardial pathological changes on the basis of cellular functional damage.

PS-MPs may cause pathological changes in the heart through the induction of cardiomyocyte pyroptosis, inflammatory cell infiltration, and mitochondrial damage (Wu et al., 2022; Zhang et al., 2022). In this study, inflammatory markers were detected in both myocardial tissue and serum, revealing that exposure to PS-MPs significantly elevated the levels of IL-1, IL-1β, and ICAM-1, as well as serum MDA level and ACP activity. Interestingly, contrary to expectations, the expression level of IL-6 in myocardial tissue decreased with the increment in PS-MPs concentration. However, it was surprising that serum level of IL-6 in myocardial tissue decreased with the increase of PS-MPs exposure concentration. IL-6 has a wide range of physiological functions in organisms, including host defense, regulation of immune cells, proliferation, and differentiation. It has been found to be involved in the pathogenesis of various chronic inflammatory diseases such as Crohn’s disease, rheumatoid arthritis, and inflammatory bowel cancer, and is also associated with atherosclerotic heart disease (Schuett et al., 2012; Ridker and Rane, 2021). However, it has been found that there are two types of IL-6 receptors in the body, the soluble IL-6 receptor (sIL-6R) -mediated trans signaling has a pro-inflammatory effect, and IL-6 can play an anti-inflammatory effect through membrane-binding IL-6 receptor (classical signaling) (Rose-John, 2018). In ApoE^−/−^IL-6^−/−^ mice with double knockout, lifelong deficiency of IL-6 may enhance the formation of atherosclerotic plaques (Schieffer et al., 2004). Thus, in atherosclerosis, baseline IL-6 levels have regulatory effects on lipid homeostasis, vascular remodeling, and plaque inflammation. Further studies are needed to determine the relationship between the decreased myocardial IL-6 level and PS-MPs injury in the present study.

The liver is the main site for metabolism and detoxification in the body. PS-MPs can affect liver lipid metabolism after entering the body (Lu et al., 2018; Luo et al., 2019; He et al., 2023). PS NPs and aPS NPs not only upregulate the expression levels of phosphatidylinositol 3-kinase (PI3K)/protein kinase B (p-AKT)/glucose transporter 4 (GLUT4) proteins in the glucose metabolism pathway in mice, but also significantly increase the expression levels of key proteins in the lipid metabolism signaling pathway, including sterol regulatory element-binding protein-1 (SREBP-1)/peroxisome proliferator-activated receptor gamma (PPARγ)/adipose triglyceride lipase (ATGL), thereby affecting glucose and lipid metabolism (He et al., 2023). Another study found that PS-MPs of different diameters mainly lead to a decrease in TG and TC levels in mouse liver, which may be related to changes in gut microbiota composition and induction of hepatic lipid disorder by polystyrene MPs (Lu et al., 2018). In this study, significant changes in blood lipid levels were observed in rats exposed to PS-MPs at a dose of 5 mg/kg/d. Specifically, there was an increase in TC levels and a decrease in TG levels. In the group exposed to PS-MPs at a dose of 50 mg/kg/day, LDL levels showed a downward trend. However, there was no significant difference in blood glucose levels. IL-6 was identified as a lipolytic factor, as the IL-6 knockout mice developed mature obesity, which was partially reversed after repeated administration of IL-6 (Wallenius et al., 2002). IL-6 has been found to induce β-oxidation through AMPK, stimulate the secretion of leptin in human retina and subcutaneous adipose tissue, thereby affecting appetite and calorie intake, and reduce the activity of lipoprotein lipase in the omentum by 56% and in the subcutaneous adipose tissue by 68% (Lehrskov and Christensen, 2019). Therefore, the decreased IL-6 levels in the present study may be related to lipid metabolism disorders caused by PS-MPs.

Non-targeted metabolomics, as an efficient and comprehensive research method for analyzing changes in body metabolites, has been widely used in toxicology research. In this study, with the increase in PS-MPs exposure concentration, metabolites such as equol, 4-HBA, oxoadipic acid, guanidinosuccinic acid and alpha-D-glucose were significantly upregulated, while ketoleucine, maltol, isobutyric acid, and oleamide were significantly downregulated. Equol is an isoflavone-derived metabolite formed by bacteria in the distal small intestine and colon from soy glycosides (Setchell and Clerici, 2010). It has the highest estrogenic and antioxidant activity (Mayo et al., 2019). Evidence from observational studies and short-term RCTs suggests that S-equol has anti-atherosclerosis effects, improves arterial stiffness, and may prevent coronary heart disease and cognitive impairment/dementia (Sekikawa et al., 2019). In addition, equol also has the potential to reduce oxidative stress in human skin and prolong the lifespan of skin cells (Lephart, 2016). Studies have shown that S-(−) equol (10-250 nM) increases the gene products of nuclear factor erythroid 2-related factor 2 (Nrf2) and Nrf2 target genes heme oxygenase-1 (HO-1) and NAD(P) H (nicotinamide adenine dinucleotide phosphate) quinone oxidoreductase 1 (NQO1), and can inhibit cell apoptosis (Zhang et al., 2013). In addition, previous studies have found that equol has an inhibitory effect on IL-6. Subedi et al. found that it can inhibit the activation of microglia and enhance the protection of neurons *in vitro* by inhibiting IL-6 (Subedi et al., 2017). In addition, I-Chian et al. found that iequol administration suppressed the expression of IL-6 and its receptor, which in turn inhibited the inflammatory response and bone erosion caused by rheumatoid arthritis in mice (Lin et al., 2016). 4-HBA is a precursor of the benzoquinone ring of CoQ (Pierrel, 2017). Coenzyme Q10 involved in many aspects of cellular metabolism, including redox homeostasis and membrane stability (Pierrel, 2017). It is primarily derived from endogenous biosynthesis and depends on the interaction of multiple enzymes in the mevalonate pathway (Bentinger et al., 2010). Studies have shown that supplementation of coenzyme Q10 can improve oxidative stress, mitochondrial dysfunction, and inflammation in various diseases (Bentinger et al., 2010). COQ2 (4-HBA-polyprenyltransferase) is an enzyme that catalyzes the synthesis of coenzyme Q10. It catalyzes the glutarylation of 4-HBA with an all-trans polyprenyl chain (Ashby et al., 1992). In cell lines with COQ2 defects, the supplementation of 4-HBA can fully restore endogenous coenzyme Q10-biosynthesis (Herebian et al., 2017). Meanwhile, a-D-glucose is a natural monosaccharide that plays a vital biological function in the body. It is one of the primary sources of cell energy, it is converted into energy through the glycolysis pathway in cells, participating in cell metabolism processes. Therefore, based on the above results, it is speculated that the upregulation of these metabolites may play a protective role in myocardium and blood vessels by inhibiting IL-6 related inflammation. This could partially explain the negative pathological changes in the myocardium and aorta in the present study.

The downregulated metabolites ketoleucine, maltol, isobutyric acid, and oleamide are common chemical substances that are widely used in different fields. Ketoleucine is an essential substance for synthesizing muscle proteins and plays an important role in the growth and repair of the body (Yee et al., 1988). Maltol is a Maillard reaction product generated from the thermal decomposition of starch or sucrose and is a natural antioxidant and preservative (Anwar-Mohamed and El-Kadi, 2007). Maltol also has anti-inflammatory and antioxidant properties, inhibiting excessive production of MDA and increasing the levels of antioxidant enzymes; and it improves oxidative stress damage by activating the phosphoinositide 3-kinase (PI3K)/protein kinase B (Akt) signaling pathway (Sha et al., 2021). Isobutyric acid (IBA) is one of the end products of intestinal microbial metabolism (Sanna et al., 2019). The level of IBA in the serum of colorectal cancer patients is significantly elevated and may be associated with colorectal cancer metastasis (Chen et al., 2023). Oleamide is an endogenous fatty acid amide with vasodilatory effects (Hoi and Hiley, 2006) and has a wide range of beneficial effects on the central nervous system (Fedorova et al., 2001). Correlation analysis of serum markers and the above metabolites in the present study showed a negative correlation between equol and serum MDA levels. These results also imply that exogenous equol supplementation may protect against PS-MPs-induced injury.

Further analysis through KEGG revealed that different concentrations of PS-MPs have an impact on the metabolic pathways of “protein digestion and absorption, arachidonic acid metabolism, butyrate metabolism, basal cell carcinoma”. As the exposure concentration of PS-MPs increases, changes also occur in metabolic pathways such as “ABC transporters, β-alanine metabolism, phenylalanine, niacin, and nicotinamide metabolism, and lysine degradation”. The main role of these metabolites is to maintain cell morphology and participate in energy metabolism. Therefore, based on the above results, it is speculated that PS-MPs may induce local inflammation and oxidative stress, leading to organ damage, through mechanisms such as disrupting cell morphology and affecting cell energy metabolism. Combined with the pathological results of this study, the changes of myocardial, aortic and blood biochemical indexes suggest that long-term accumulation of high-dose PS-MPs may have potential damage to the cardiovascular system. However, the activation of metabolites such as equol and 4-HBA in rats may play a protective role in the cardiovascular system by anti-oxidation and inhibiting inflammatory response. This study provides a new idea and theoretical basis for further exploring the cardiovascular toxicity of PS-MPs and its prevention and treatment.

## 5 Conclusion

In summary, the impact of PS-MPs on the cardiovascular system may be a process of low-toxicity slow damage, with myocardial cell inflammation and oxidative stress as the main mechanisms. Combined with the changes in biochemical indicators, we found that IL-6 balance plays an important role. Untargeted metabolomics results showed that activation of some metabolites, such as equol and 4-HBA, had protective effects on the cardiovascular system. Supplementation or regulation of metabolite production may contribute to anti-PS-MPs damage.

## Data Availability

The raw data supporting the conclusion of this article will be made available by the authors, without undue reservation.
